# Axial Diffusivity of the Corona Radiata at 24 Hours Post-Stroke: A New Biomarker for Motor and Global Outcome

**DOI:** 10.1371/journal.pone.0142910

**Published:** 2015-11-12

**Authors:** Eric Moulton, Mélika Amor-Sahli, Vincent Perlbarg, Christine Pires, Sophie Crozier, Damien Galanaud, Romain Valabregue, Marion Yger, Flore Baronnet-Chauvet, Yves Samson, Didier Dormont, Charlotte Rosso

**Affiliations:** 1 Centre de Recherche de l'Institut du Cerveau et de la Moelle épinière, Paris, France; 2 UPMC Paris 6, Inserm, U1127, CNRS, UMR 7225, Paris, France; 3 CONAM, UPMC Paris 6, Inserm, U1127, CNRS, UMR 7225, Paris, France; 4 AP-HP, Department of Neuroradiology, Pitié-Salpêtrière Hospital, Paris, France; 5 INSERM U1146, CNRS UMR7371, laboratoire d’imagerie biomédicale, Sorbonne université, UPMC université, Paris 60 UMCR2, Hôpital de la Pitié-Salpêtrière, Paris, France; 6 IHU-A-ICM, Bioinformatics/Biostatistics Plateform, Paris, France; 7 APHP, Urgences Cérébro-Vasculaires, Hôpital Pitié-Salpêtrière, Paris, France; 8 COGIMAGE, Centre de Recherche de l'Institut du Cerveau et de la Moelle épinière, Paris, France; 9 Centre de Neuro-Imagerie de Recherche, http://cenir.org, Hôpital Pitié-Salpêtrière, Paris, France; Brighton and Sussex Medical School, UNITED KINGDOM

## Abstract

Fractional anisotropy (FA) is an effective marker of motor outcome at the chronic stage of stroke yet proves to be less efficient at early time points. This study aims to determine which diffusion metric in which location is the best marker of long-term stroke outcome after thrombolysis with diffusion tensor imaging (DTI) at 24 hours post-stroke. Twenty-eight thrombolyzed patients underwent DTI at 24 hours post-stroke onset. Ipsilesional and contralesional FA, mean (MD), axial (AD), and radial (RD) diffusivities values were calculated in different Regions-of-Interest (ROIs): (1) the white matter underlying the precentral gyrus (M1), (2) the corona radiata (CoRad), (3) the posterior limb of the internal capsule (PLIC) and (4) the cerebral peduncles (CP). NIHSS scores were acquired at admission, day 1, and day 7; modified Rankin Scores (mRS) at 3 months. Significant decreases were found in FA, MD, and AD of the ipsilesional CoRad and M1. MD and AD were also significantly lower in the PLIC. The ratio of ipsi and contralesional AD of the CoRad (CoRad-rAD) was the strongest diffusion parameter correlated with motor NIHSS scores on day 7 and with the mRS at 3 months. A Receiver-Operator Curve analysis yielded a model for the CoRad-rAD to predict good outcome based on upper limb NIHSS motor scores and mRS with high specificity and sensitivity. FA values were not correlated with clinical outcome. In conclusion, axial diffusivity of the CoRad from clinical DTI at 24 hours post-stroke is the most appropriate diffusion metric for quantifying stroke damage to predict outcome, suggesting the importance of early axonal damage.

## Introduction

Diffusion Tensor Imaging (DTI) is a Magnetic Resonance Imaging (MRI) technique allowing for quantification of the integrity of nerve fibers in the brain [1–3]. Four diffusion indices computed from the diffusion tensor are commonly used to quantify neuronal integrity: (1) Fractional Anisotropy (FA), a measure of the percent of the diffusion tensor associated with anisotropic movement, (2) Mean Diffusivity (MD), a measure of the average magnitude of water diffusion in three dimension, closely related to the Apparent Diffusion Coefficient (ADC), (3) the principle eigenvalue, also referred to as Axial Diffusivity (AD), and (4), the average of the two remaining perpendicular eigenvalues, also referred to as Radial Diffusivity (RD).

In the context of stroke imaging, DTI has been used as an additive measure for motor prediction in ischemic stroke patients by examining the severity of damage to motor related paths such as the cortical spinal tract (CST) [4]. In initial studies investigating damage to the CST, FA was the primary diffusion parameter for characterizing of neuronal integrity. Indeed, decreased FA in the ipsilesional CST at the sub-acute and chronic stages is a strong correlate of long-term outcome [4–6]. In the few studies that scanned ischemic stroke patients within the first week of stroke onset, however, FA performs less well in quantifying stroke damage and future motor recovery [6–8]. One proposed explanation for this is that at acute time scales, cellular mechanisms, such as cytotoxic edema, are at play in the infarct and have different dynamic effects on axial and radial diffusivities [9–10] and can thus lead to pseudonormal FA values. Moreover, in these studies, patients are rarely scanned at the same time point. Patient inclusion in the crossover period of the sub-acute and acute stages may thus result in inconsistencies between diffusion metrics and motor outcome prediction. Obtaining useful data in the clinical setting at the earliest time point is of crucial interest, especially in the context of thrombolytic treatments, in order to predict not only subacute motor outcome, but also long-term global outcome. The present study addresses these concerns by assessing ischemic stroke damage with DTI at 24h post-stroke. The global aims were to determine an early biomarker associated with motor outcome at day 7 and global outcome at 3 months. The specific aims were (i) to compare diffusion metrics in the affected *vs*. non affected hemisphere along regions-of interest (ROIs) of the corticospinal tract, (ii) to determine which diffusion metric in which ROI correlates most strongly with 7-day motor outcome and 3-months global outcome, and (iii) to determine the accuracy of the best diffusion metric in motor and global outcome prediction. In order to facilitate this procedure’s entry in the clinical setting, an automated pipeline was implemented to warp FA, MD, and eigenvalue maps in a normalized space for all patients.

## Materials and Methods

### Patients

Sixty-six patients were screened for the study and were scanned from September 1, 2013 until September 1st, 2014 at the Urgences Cérébrovasculaires at the Hôpital de la Pitié Salpêtrière. The local ethics committee (Paris VI IRB) approved the study, and oral consent was given (instead of written) since all imaging and clinical data were generated during the routine clinical workup of the patients in our stroke center. Oral consent was documented in each patient's medical file, and the local ethics committee approved this procedure.

Inclusion criteria were (1) MRI-demonstrated ischemic stroke of the carotid territory, (2) thrombolysis treatment within 4.5 hours after stroke onset, and (3) follow-up MRI access at 24 hours post-stroke. Thrombolytic treatment was administered according to the American Stroke Association and the European Stroke Organization guidelines (0.9 mg/kg, maximal dose 90 mg) [11]. Of 66 patients thrombolyzed during the study period, 28 underwent the 24-hour follow-up diffusion tensor imaging (DTI) scans. No significant differences between the included and the excluded populations were found for age (p = 0.24), National Institute of Health Stroke Scales (NIHSS) scores at admission (p = 0.29), or delay of thrombolysis treatment (p = 0.12). Patients had NIHSS scores taken at admission, the day of scanning (24 hours post stroke), and at 7 days post stroke. NIHSS scores used in the analysis were the following acquired on day 7: NIHSS items 5 and 6 individually, for the upper limb (UL) and lower limb (LL) respectively (only scores for the contralesional side were taken), and the sum of both for a total motor score (MOT). A modified Rankin Scale (mRS) score was obtained at 3 months following the patients’ strokes. NIHSS scores and mRS were evaluated by certified stroke neurologists. While it is known that recovery of the upper limb continues after day 7 [12], the NIHSS scores were used to investigate which metric(s) were able to reflect sub-acute damage. The mRS scores, contrarily, were included as a global outcome measure to assess the predictive value of the diffusion metric(s) acquired 24 hours post-stroke. Secondary prevention treatment and rehabilitation were adapted from clinical work up and the disability of the patients; on average, patients received 2–3 hours of physical therapy per week until the 3-month assessment.

### Image Acquisition

Twenty-four hours after admission, patients underwent a follow-up MRI with a General Electric 3T MRI scanner. The following sequences were performed: (1) Diffusion Weighted Imaging, (2) Diffusion Tensor Imaging (DTI), (3) FLAIR, (4) T2*, and (5) MR angiography (MRA). The Echo Planar Image DTI sequence was performed with an 8-channel coil. Imaging parameters were as follows: TR = 12000 ms, TE = 80.1 ms, Voxel size = 3x3x3 mm^3^. The DTI sequence comprised 2 non-diffusion-weighted images (b = 0 s/mm^2^) followed by diffusion-weighted images in 30 non-collinear directions (b = 1000 s/mm^2^).

### Image Processing

After scanning, all volumes were inspected visually for irreparable artifacts due to susceptibility artifacts or head movements. All faulty volumes were removed before preprocessing (no more than 6 volumes were removed for any given patient). Diffusion weighted images were preprocessed using the FDT Diffusion Toolbox in FSL (http://fsl.fmrib.ox.ac.uk/fsl/fslwiki/). Preprocessing consisted of (1) realignment of the diffusion weighted volumes to the average of the two non-diffusion-weighted images. (2) correction for eddy-currents and head movements, and (3) estimation of a single-tensor model to calculate Fractional Anisotropy (FA) a measure of the percent of the diffusion tensor associated with anisotropic movement, Mean Diffusivity (MD), a measure of the average magnitude of water diffusion in three dimension, closely related to the Apparent Diffusion Coefficient (ADC), tensor eigenvectors and the corresponding eigenvalues. In this paper, we refer to the principle eigenvalue as Axial Diffusivity (AD) and the average of the two remaining perpendicular eigenvalues as Radial Diffusivity (RD).

To evaluate diffusion metrics within predefined ROIs designed in MNI standard space (MNI152 space), FA maps were non-linearly registered to the 1x1x1 mm^3^ MNI standard space image using Tract-Based Spatial Statistics (TBSS) [13]. This procedure limits the possible misalignment problems between subjects by projecting the locally maximal FA values onto the FA skeleton template representing the centers of the main white matter tracts. Once the registration was complete, MD, AD, and RD maps were projected onto the FA skeleton by applying the same non-linear transformation. Finally, average diffusion values were computed within predefined ROIs as described by van der Eerden et al. [14]. The method consists of a selection of regions of the ICBM-DTI-81 white-matter labels atlas proposed by Mori et al. [15]. The ROI of the corona radiata of this atlas was modified to limit rostral and caudal projections unrelated to motor function: an anterior limit of MNI plane y = 3mm and a posterior limit of MNI plane y = -35mm were used as cut-off points. We chose to examine the part of the corona radiata where projection fibers are concentrated. The superio-inferior boundaries were not modified from the atlas of Mori et al.[15], and already used for assessing patients [16]. The anterior boundary was the premotor cortex, as it has been well described that corticofugal fibers from this region travel with the fibers originating from the primary motor cortex and participate in motor control [17]. The posterior boundary was the parietal cortex since pre- and post-central regions corresponding to the hand area of the motor-sensory homunculus are connected in order to improve sensorimotor integration [18]. An additional analysis was performed to investigate the orientations of the tensors of the corona radiata in order to determine the fiber populations located within the CoRad ROI (see S1 Text). The CoRad ROI seems to capture mainly descending projection fibers from the motor cortices. Diffusion changes in this region would therefore seem to reflect damage to this fiber population. Additionally, an extra hand-drawn ROI was added in the analysis to include the underlying white matter of the precentral gyrus (MNI coordinates: 52mm ≤ z ≤ 59mm).

**Fig 1:**
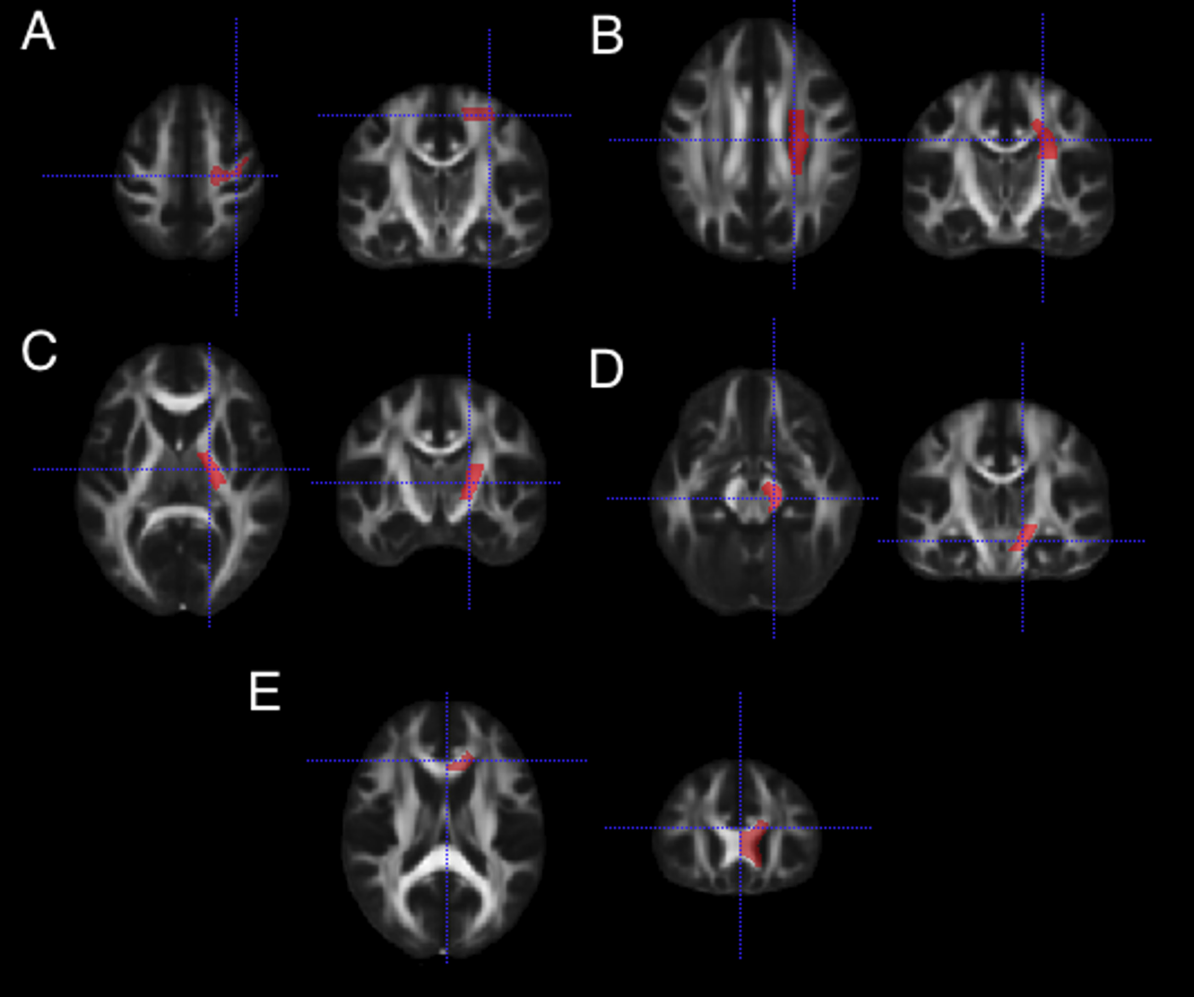
Regions-of-Interest for extraction of diffusion parameters. A preview of the Regions-of-Interest (in red) used in the analysis overlaid on the FMRIB58 fractional anisotropy standard space image. (A) White matter underlying the pre-central gyrus. (B) Corona Radiata. (C) Posterior Limb of Internal Capsule (D) Cerebral Peduncles (E) Genu of the Corpus Callosum.

For this study, only ROIs characterizing different levels of descending corticospinal fibers were retained. The final list of ROIs consisted of (1) sub-cortical white matter of the precentral gyrus (M1), (2) Corona Radiata (CoRad), (3) Posterior Limb of the Internal Capsule (PLIC), and (4) Cerebral Peduncles (CP). Separate ROIs were used for the ipsilesional and contralesional hemisphere. A control region was taken outside the corticospinal tract, in the genu of the corpus callosum. Previews of the ROIs used in this analysis can be seen in Fig 1. The ratios of diffusion metrics were computed by dividing the average value in the ipsilesional ROI by the average value of its corresponding contralesional ROI (given as rFA, rMD, rAD, and rRD).

Two reviewers manually segmented infarct lesions on each patient's diffusion-weighted images (b = 1000s/mm^2^) from an acquisition separate from the 30-direction DTI scan. A two-way intraclass correlation coefficient was computed for lesion volume to assess inter-rater reliability. The intraclass correlation coefficient was 0.991 (95% confidence interval = 0.981–0.996, p<0.0001). We constructed a probability map of the infarcts by non-linearly warping all lesion masks to MNI space by a two-step process. First, each patient’s diffusion-weighted images from the first acquisition were coregistered to the FA maps obtained from the DTI sequence using a rigid transformation with 6 degrees of freedom using FMRIB’s Linear Image Registration (FLIRT) in FSL [19–20]. Second, warp maps obtained from the TBSS transformation of the FA maps to MNI space were applied to the diffusion- weighted images and segmentations with the aforementioned rigid transformation matrix as a pre-matrix using FMRIB’s Non-linear Registration Tool (FNIRT) [21]. The segmentation maps of patients who had lesions on the right were flipped, so all lesions were in the left hemisphere. Binary maps were finally superimposed to produce an infarct probability overlap map. The percentage of overlap of the patients’ lesions and each ROI was calculated (infarct-ROI overlap). This overlap corresponds to the part of the ROI that is directly damaged by the infarct.

### Statistical Analysis

The descriptive statistics include the mean and standard deviation (SD), the median and interquartile range (IQR), and range. Significant differences in diffusion metrics on the affected *vs*. unaffected side were tested with paired Wilcoxon signed-rank tests. Significant correlations between ratios of average diffusion metrics and clinical scores at day 7 post-stroke and at 3 months were tested with Spearman's rank correlation tests. Lesion volume and age were also included in the correlation analysis. Statistical significance is set at p≤0.01 in order to account for multiple comparisons of the five ROIs. In order to see interdependent relations, significant correlations are further tested with partial correlations using lesion volume as a confounding variable. Ninety-five percent confidence intervals (95% CI) for Spearman correlations and partial correlations were obtained from bootstrapping using 10,000 replications.

Next, we created two groups: (1) those patients who had complete recanalization and (2) those with incomplete or no recanalization. Recanalization was considered on a two-item scale: (1) complete recanalization, (2) no complete recanalization defined as partial or minimal flow-related signal in the region of the arterial clot or persistent occlusion. Between both groups, we compared the values of the diffusion metrics of the ROI which will be the most correlated to clinical scores as well as the infarct-ROI percent overlap of the same ROI with Wilcoxon signed-rank tests. In doing so, we investigated if early recanalization or prolonged ischemia (i.e. no recanalization) influenced both the severity of ischemia (assessed by diffusion decrease in the ROI) and the extent of the infarct (assessed by the percentage of overlap between the infarct lesion and the ROI) or just one of these two mechanisms.

Finally, clinical scores for the UL and the mRS were dichotomized into good and poor outcomes to construct Receiver-Operator Characteristic curves (ROC) to predict good outcome with the diffusion metrics most correlated to clinical scores. We chose to include only upper limb extremity outcome from the NIHSS scores because it is one of the most difficult items to predict, and clinical variables only moderately predict motor recovery [12]. At day 7, an upper limb NIHSS motor score of 0 or 1 was considered as “good” outcome. Two ROC analyses were performed for the mRS at 3 months. The first considered very good outcome as a mRS ≤ 1, and the second considered good outcome as a mRS ≤ 2. These two specific cutoff points were chosen as they correspond to distinct clinical outcomes. Patients with a mRS ≤ 1 have no or minor deficits, able to carry out all previous Activities of Daily Living (ADLs), whereas those with a mRS = 2 have noticeable deficits that affect certain ADLs but are able to live independently. Finally, those with a mRS > 2 are unable to carry out ADLs without some assistance. ROC curves are plotted as (1-specificity) vs. sensitivity. Thresholds representing an optimal compromise between specificity and sensitivity are those of the point on the ROC curve closest to the top-left corner. In addition, we plotted the Positive Predictive Values (PPV) and Negative Predictive Values (PPV) for good motor outcome, as well as for good and very good global outcome as a function of the most correlated diffusion metric ratios. We explore the limits of these models by presenting the thresholds up to 100% PPV and NPV in order to make certain predictions and circumvent compromises for “optimal” thresholds. A diffusion measure with a PPV of 100% is indicative of certain good outcome, whereas a diffusion measure with a NPV of 100% is indicative of certain poor outcome. This idea of a “point of no return” is of strong clinical utility for the allocation of resources for rehabilitation strategies [22]. All statistics were computed using the R-programming language and packages [23–27].

## Results

### Patients

Descriptive characteristics for the patient population are given in Table 1. Seventeen (61%) were female, 22 (79%) had lesions of the left hemisphere, 21 (75%) had an intracranial occlusion visible on the pre-thrombolysis MRA of which 17 (61%) had complete recanalization after thrombolysis treatment. One patient (4%) had an uninterpretable recanalization. At three months, two patients had missing data for the mRS. One patient had died from cardiac complications and a second had a recurrent stroke. Of the remaining 26 patients, 10 (38%) had mRS scores of 1 or lower, 12 (47%) had mRS scores of 2 or lower, 10 (38%) had mRS scores between 3 and 5, and 4 (15%) had deceased within the 3 months of their stroke (mRS = 6).

**Table 1:** Characteristics of the included patients.

Measure	Mean	SD	Median	IQR	Range
Age (years)	68.5	16.9	68.4	54.0–83.0	36–95
Delay Thrombolysis (min)	163.2	78.0	135.0	121.7–192.7	65.0–390.0
Lesion Volume at Day 1 (cm^3^)	41.7	84.4	8.1	2.8–56.0	0.1–418.6
**Admission NIHSS**					
Total	15.4	7.4	14.5	8.0–22.3	5–26
MOT	4.6	3.1	6.0	1.8–7.0	0–8
UL	2.4	1.6	3.0	0.8–4.0	0–4
LL	2.3	1.6	3.0	1.0–4.0	0–4
**24-H NIHSS**					
Total	9.7	9.3	5.0	2.0–18.0	0–25
MOT	3.0	3.5	0.5	0.0–7.0	0–8
UL	1.5	1.9	0.0	0.0–4.0	0–4
LL	1.4	1.7	0.0	0.0–3.0	0–4
**DAY 7 NIHSS**					
Total	7.5	8.2	4.0	0.0–13.5	0–26
MOT	2.2	3.2	0.0	0.0–4.5	0–8
UL	1.2	1.7	0.0	0.0–2.5	0–4
LL	1.0	1.6	0.0	0.0–2.0	0–4

UL = Upper Limb, LL = Lower Limb, MOT = UL+LL. SD = standard deviation. IQR = interquartile range.

**Fig 2:**
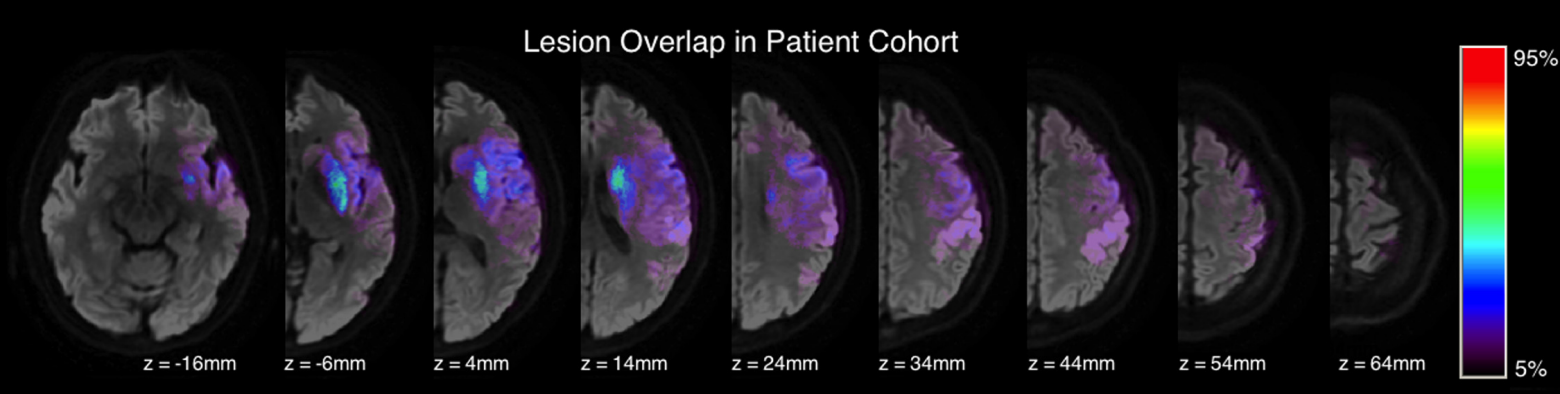
Infarct probability map of the patient cohort. Infarct probability map overlaid on a diffusion-weighted volume of a selected patient. Color map corresponds to the percentage of patients with infarcted tissue in a given voxel. Z-coordinates are in MNI space.

The infarct probability map is shown in Fig 2. S1 Table contains the infarct-ROI percent overlap for each patient of the cohort. The highest overlap occurred in the putamen and in the periventricular white matter. A total of 23 patients (82%) had infarcts in the CoRad with median 2.6% overlap (IQR: 0.2–11.0), 15 (54%) were in the posterior limb of the internal capsule with median 0.1% overlap (IQR: 0.0–9.7), and 6 (21%) were in the underlying white matter of the precentral gyrus with median 0.0% overlap (IQR: 0.0–0.0). In 4 patients, the infarct did not overlap with any of the predefined ROIs, and, as expected, in these carotid territory strokes, the infarct never overlapped with the CP ROI. As no infarcts were in the CP ROI, a Kruskal-Wallis test was performed with the percent overlaps of the PLIC, CoRad, and M1 ROIs to see if the distribution of infarct damage was similar between these three ROIs. The test revealed significantly unequal damage between ROIs (p < 0.001). Wilcoxon signed-ranked tests showed that there was no difference between the infarct-ROI percent overlap in the PLIC and CoRad (p = 0.1), yet the infarct-ROI percent overlap of M1 was lower than that of the PLIC (p < 0.01) and CoRad (p < 0.0001).

### Comparison of the Diffusion Metrics Between Affected and Non-Affected Sides

Significant decreases in the MD and AD occurred in CoRad, PLIC, and M1 ROIs. Decreases in FA occurred in the CoRad and M1. We did not detect significant abnormalities in RD values. There were no significant differences in diffusion metrics in the cerebral peduncles. As for the control region, only differences in MD reached statistical significance (p = 0.01); however, this may be due to mass effects caused by the infarct. The values of diffusion metric ratios are given in Table 2.

**Table 2:** Ratios of diffusion metrics in the four Regions-Of-Interest and control region.

ROI	rFA	rMD	rAD	rRD
CP	0.99 (0.98–1.02)	0.98 (0.94–1.02)	0.98 (0.95–1.03)	0.98 (0.94–1.01)
PLIC	0.97 (0.95–1.02)	0.98 (0.94–1.00)*	0.97 (0.94–1.00)**	0.99 (0.93–1.02)
CoRad	0.98 (0.94–1.01)*	0.98 (0.92–0.99)*	0.96 (0.91–0.99)***	0.99 (0.93–1.02)
M1	0.95 (0.91–1.00)*	0.97 (0.95–0.99)**	0.95 (0.92–0.99)***	1.00 (0.95–1.03)
gCC	0.99 (0.97–1.01)	0.99 (0.98–1.00)*	0.99 (0.97–1.01)	0.99 (0.98–1.01)

Ratios given as Median (IQR). Significant differences between contra and ipsilesional sides given as

*p≤0.01

**p≤0.001

***p≤0.0001. CP = Cerebral Peduncles, PLIC = Posterior Limb of the Internal Capsule, CoRad = Corona Radiata, M1 = Underlying White Matter of the Precentral Gyrus, gCC = genu of the Corpus Callosum.

### Correlation Analysis between Diffusion Metrics and Clinical Scores

After correction for multiple comparisons, the only diffusion metrics correlated with all clinical scores were the rAD and rMD of the CoRad, given as CoRad-rAD and CoRad-rMD respectively. The lower the CoRad-rAD and the CoRad-rMD were, the poorer the outcome was. The infarct volumes also correlated with all clinical scores. Table 3 contains a summary of the global and partial correlations for the CoRad-rAD, CoRad-rMD, and volume with clinical scores. The rRD and rFA of any ROI did not correlate with the clinical scores. Age was not correlated with any clinical score, and neither were the diffusion metrics of the control region (genu of the corpus callosum).

**Table 3:** Global and partial correlations between diffusion metrics of the corona radiata, infarct volume, and outcome.

**Global Correlations**				
	rAD		rMD	Volume
	ρ (95%CI)		ρ (95%CI)	ρ (95%CI)
UL	-0.644***		-0.514**	0.466*
	(-0.923;-0.396)		(-0.856;-0.200)	(0.148;0.805)
LL	-0.608***		-0.490**	0.394*
	(-0.886;-0.353)		(-0.853;-0.149)	(0.074;0.736)
MOT	-0.654***		-0.525**	0.473*
	(-0.937;-0.400)		(-0.879;-0.198)	(0.157;0.812)
mRS	-0.698***		-0.647***	0.585**
	(-0.923;-0.505)		(-0.935;-0.390)	(0.324;0.873)
**Partial Correlations**				
	rAD \ rMD	rMD \ rAD	rAD \ Volume	Volume \ rAD
	ρ (95%CI)	ρ (95%CI)	ρ (95%CI)	ρ (95%CI)
UL	-0.507**	0.257	-0.516**	0.138
	(-0.806;-0.260)	(-0.050;0.647)	(-0.810;-0.223)	(-0.316;0.570)
LL	-0.458*	0.219	-0.505**	0.055
	(-0.745;-0.230)	(-0.094;0.623)	(-0.800;-0.214)	(-0.358;0.453)
MOT	-0.513**	0.257	-0.527**	0.140
	(-0.798;-0.278)	(-0.041;0.643)	(-0.827;-0.227)	(-0.302;0.560)
mRS	-0.349	0.051	-0.512**	0.228
	(-0.808;0.061)	(-0.449;0.595)	(-0.836;-0.214)	(-0.192;0.649)

Spearman rank correlation coefficients ρ (95% Confidence Interval) for Spearman correlations between the axial (rAD), mean (rMD) diffusivity ratios in the corona radiata and volume with clinical scores at day 7 and 3 months. UL = Upper Limb, LL = Lower Limb, MOT = Total Motor Score, mRS = modified Rankin Scale. Partial correlations between a predictive measure (measure 1) and a clinical score (left rows) while controlling for another predictive measure (measure 2) are represented with the notation measure 1 \ measure 2 (columns). For example, the cell where column rAD\rMD intersects row UL corresponds to the partial correlation of rAD with UL while controlling for rMD.

*p≤0.05

**p≤0.01

***p≤0.001

Due to the linear dependence of MD on AD, the CoRad-rMD, and CoRad-rAD were further included in a partial correlation to see if information from the perpendicular eigenvalues contributed significantly to correlations of rMD. Partial correlation analyses revealed that rAD remained significantly correlated with the motor items of the NIHSS when corrected for rMD with a similar trend for the mRS at three months (p = 0.07). Conversely, when corrected for rAD, the rMD was no longer significantly correlated with any clinical scores.

The strongest independent predictor of the clinical scores was therefore the CoRad-rAD. It therefore entered a partial correlation with total infarct volume. The CoRad-rAD remained significantly correlated with all clinical scores once adjusted for volume. The volume was no longer correlated with clinical scores when adjusted for the CoRad-rAD.

### Effect of Recanalization on Diffusion Changes and Infarct Volume

The CoRad-rAD was found to be significantly different in patients with complete recanalization *vs*. those without (median (IQR): 0.99 (0.96–0.99) *vs*. 0.86 (0.71–0.94), p < 0.001). On the other hand, there was no significant difference in ROI-infarct percent overlap in the CoRad (2.3% (0.1%-8.0%) vs. 4.7% (1.6% - 28.4%), p = 0.2). While we cannot draw any conclusions from this non-significant result, it seems to suggest that in non-recanalized patients, extent of infarction within the ROI was nearly the same as those of recanalized patients.

### ROC Analysis to Predict Sub-Acute Upper Limb Motor and Long-Term Global Outcome

Table 4 contains a summary of the ROC analyses. A ROC Curve analysis to predict upper limb scores of 0–1 at day 7 yielded a model with an optimal threshold of CoRad-rAD = 0.945 with a specificity of 78% and a sensitivity of 83%. For the mRS ≤ 1 at 3 months, the optimal threshold was CoRad-rAD = 0.960 with a specificity of 79% and a sensitivity of 100%. For the mRS ≤ 2 at 3 months, the optimal threshold remained CoRad-rAD = 0.960 with the same specificity of 79% and a lowered sensitivity of 83%.

**Table 4:** Receiver-Operator Curve (ROC) models for CoRad-rAD.

	Optimal rAD	AUC	Acc (%)	Spec (%)	Sens (%)	rAD for 100% NPV	rAD for 100% PPV
UL ≤ 1	0.945	0.89	81	78	83	0.870	0.990
mRS ≤ 1	0.960	0.93	88	79	100	0.960	0.990
mRS ≤ 2	0.960	0.88	81	79	83	0.900	0.990

ROC analyses presented are for an upper limb (UL) score of ≤ 1 at day 7 and a modified Rankin Scale (mRS) score of ≤ 1 and ≤ 2 at three months. The optimal threshold of CoRad-rAD (compromise between specificity and sensitivity) for each model is given with the associated area under the curve (AUC), accuracy (Acc), specificity (Spec), and sensitivity (Sens). For each model, the upper bound of 100% negative predictive power (NPV) and the lower bound of 100% positive predictive value (PPV) are given.

**Fig 3:**
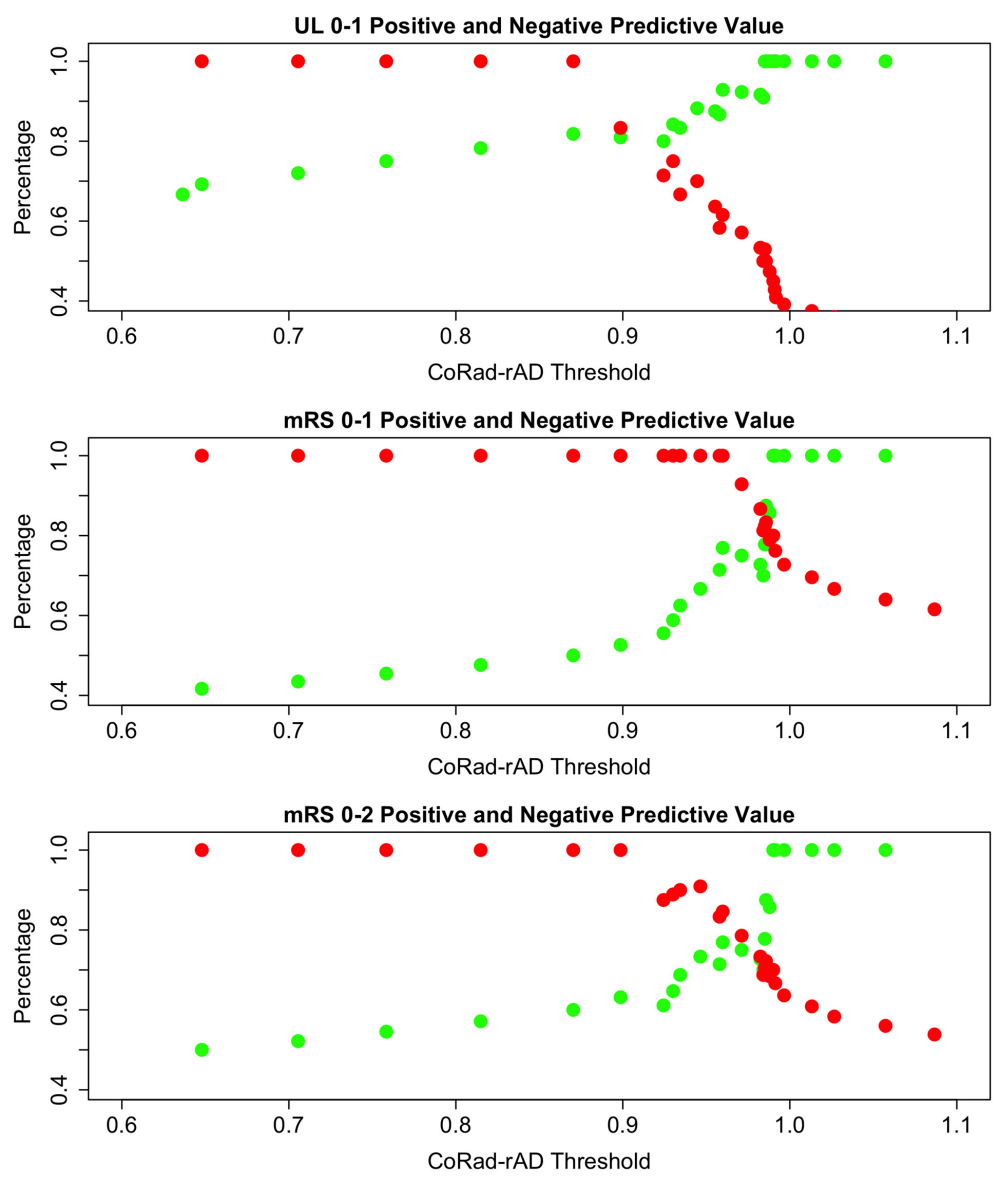
Positive and Negative Predictive Values for good outcome as a function of CoRad-rAD values. The positive predictive values (PPV in green) correspond to the CoRad-rAD values above the threshold (x-axis). The negative predictive values (NPV in red) correspond to the CoRad-rAD values below the threshold. (A) Good outcome is assessed by NIHSS item 5 for upper limb (UL) scores 0–1 at day 7 post-stroke. (B) Very good outcome is assessed by mRS ≤ 1 at 3 months post-stroke. (C) Good outcome is assessed by mRS ≤ 2.

Fig 3 shows the quantitative relationships between CoRad-rAD threshold values and outcome. The PPV for good outcome for all models reached 100% for CoRad-rAD = 0.990 indicating that normal CoRad-rAD was always associated with good outcome. Conversely, the upper bound 100% NPV thresholds for all three models were different, depending on the specific outcome. In particular, for upper limb function, no patient with a CoRad-rAD lower than 0.870 had a good outcome. With respect to the mRS, all patients with a CoRad-rAD asymmetry ≥ 4% had deficits affecting their ADL (mRS > 1), whereas a more pronounced CoRad-rAD asymmetry of ≥ 10% corresponded to dependency on exterior aid in all cases (mRS > 2).

## Discussion

In this study, we set out to find DTI-derived correlates of infarct damage at the acute stage to predict motor and global outcome. We found significant differences in the PLIC, CoRad and M1. MD and AD ratios were more sensitive to detect these changes at the acute phase, and rAD extracted from the corona radiata was independently correlated with outcome. Moreover, the rAD in the corona radiata could help to predict either the subacute motor or the chronic global outcome using a similar threshold and with great accuracy.

### Comparison of the Diffusion Metrics Between Affected and Non-Affected Sides

Significant differences in MD, and AD were seen in the underlying white matter of the precentral gyrus, the corona radiata, and the posterior limb of the internal capsule, but not in the cerebral peduncles localized in the non-ischemic vertebro-basilar territory. This first observation is consistent with the dynamics of acute neuronal damage from stroke. Several studies have shown that diffusion changes happen first in the infarct and later extend to distal parts of the nerve fibers in the following days through Wallerian degeneration [9,28]. The fact that MD, and AD are significantly different between the affected *vs*. the non-affected side, while RD is not, could be explained by early stroke physiopathology. At the acute stage (~24h post-stroke), different changes of the diffusivities occur at different rates. First, axonal structures degrade due to cell necrosis, decreasing axial diffusivity. Additionally, cellular mechanisms at early time points (i.e. within the first 24 hours of stroke onset) such as cytotoxic edema have been suggested to decrease radial diffusivity as a result of cellular swelling and an increase of extracellular tortuosity [3,10]. In the subsequent post-stroke period (beyond 24 hours of stroke onset), the effects of cytotoxic edema diminish, and myelin sheets begin to degrade, leading to "leaky" axons and thus an increase in radial diffusivity [10,28]. Because our data were obtained in a time frame in which radial diffusivity switches dynamics (from swelling due to cytotoxic oedema to myelin degradation), the radial diffusivity may have begun approaching quasi-normal values, resulting in no significant differences in RD. On average, RD decreased in our patients yet did not reach statistical significance. This is in line with Bhagat et al.’s report of a greater reduction in AD than RD 9 to 37 hours after stroke onset [29]. However, the pathophysiology of this AD decrease is not entirely clear.

### rAD of the Corona Radiata and Clinical Outcome

The CoRad-rAD survived corrections by the CoRad-rMD in a partial correlation analysis for motor and global outcome, suggesting that the observed MD decreases were being driven by AD decreases. CoRad-AD decrease was therefore the only independent predictor of one week motor deficit and 3 month mRS. This result also provides evidence that to best study stroke damage at 24 hours post-stroke, a tensor model to calculate AD will yield more precise information than simple DWI acquisitions and an ADC computation. In a recent study, Groisser also reported in 10 patients that AD decrease in the CST 3–7 days after stroke was the best predictor of motor outcome [8]. Here the CoRad-rAD at 24h post-stroke was a surprisingly sensitive biomarker: all patients with normal values (≥ 0.99) had excellent functional outcome (mRs 0–1) whereas all patients with values ≤ 0.96 had mRS scores ≥ 1 and all patients with values ≤ 0.90 had mRS scores ≥ 2. In other words, at 24 hours after onset, even small asymmetries in CoRad-AD (4 to 10%) had a major impact on the functional outcome at 3 months.

We also address the significance of the corona radiata as the most correlated ROI for motor and global outcome prediction. The corona radiata is known to be a crossroad between projection, transcallosal, and association tracts. It seems likely that this region is associated with global outcome as it creates some type of multiple disconnection syndrome in several main fasciculi. An association between damage to the corona radiata and the modified Rankin scale has also been found in a voxel-based analysis study [30]. In some studies, alternate descending motor pathways and their implication in motor stroke recovery have been highlighted [31–32]. They showed that while decreased anisotropy of descending fibers from the primary motor cortex had the strongest effect on functional hand outcome, damage to alternate motor pathways also added predictive value to hand motor outcome. Moreover, while the highest number of lesions fell in the CoRad ROI, there was no significant difference between infarct-ROI percent overlaps of the CoRad and PLIC. Nevertheless, the CoRad was the only ROI in which diffusion changes correlated with clinical outcome, despite the PLIC’s known involvement in motor prediction [33]. This finding supports the specificity of the corona radiata to patient independency due to its mediation of various functions that go beyond motor control.

Second, we discuss the relevance of axial diffusivity in quantifying neuronal damage from stroke. In the majority of studies where damage to the CST was correlated with motor outcome at acute or chronic stages, fractional anisotropy appeared to be the metric of choice [4]. FA measures the directionality of water motion and is a more complex function of AD and RD. In chronic stroke, FA changes reflect direct ischemic injury and secondary Wallerian degeneration since it is thought to detect both disintegration of axonal structures and demyelination [34]. Here FA was decreased in the CoRad and M1 but did not correlate with the clinical outcome. This is in line with the data of the literature as the predictive value of FA changes is clearly established in chronic and sub-acute strokes [4] but not during the first few days after stroke onset [6–8]. This issue has been discussed in several papers and has been related to dynamic cellular processes of individual diffusivities [3,9,28]. In Groisser et al.’s paper, the FA in the CST at two months post-stroke but not at the initial 3–7 days post-stroke was predictive of outcome at 6 months.

### The Role of Infarct Size vs Diffusion Abnormalities in the ROIs

One concern of measuring diffusion properties at such an early stage is whether or not the differences are uniquely and directly attributable to the infarct lesion or also to early remote effects. It’s expected that the infarct lesion probably, for the most part, directly explained these changes since we are at an early time point. For example, the four patients with more than 20% of infarct-ROI overlap in the CoRad had extremely low CoRad-rAD values ranging from 0.39 to 0.76. However, on average, the overlap between the infarct and the CoRad ROI was rather small. Indeed, 50% of patients had less than 2.6% of infarct-ROI overlap in the CoRad (<0.2cc of infarcted tissue). Nevertheless, clinically relevant CoRad-AD decreases were found in patients with none or minimal overlap, and a significant correlation was found between three-months outcome and CoRad-rAD in patients with a infarct-ROI overlap < 2.6% (ρ: -0.600 95%CI: -0.892;-0.048, p = 0.02); the infarct-ROI overlap therefore cannot entirely explain the patients' deficits. First, we found that the infarct-ROI overlap was not significantly different between patients with complete *vs*. incomplete or unsuccessful recanalization, suggesting that these two groups shared similar damage in the ROIs. Second, we found that the CoRad-rAD between these two patient groups was significantly different, providing evidence that a failed or impartial recanalization leads to a longer-lasting subthreshold ischemia, resulting in lesions to white matter that extend beyond the infarct itself. For instance, three patients with incomplete recanalization and minimal infarct in the CoRad (0%, 2.3%, and 2.5% overlap) had also clinically relevant rAD decreases (CoRad-rAD < 0.93) and consequently poor outcome (mRS > 2). These cases support the idea that some type of ischemic white matter injury may occur outside of the infarct area. This observation would be consistent with the recent finding of Tisserand et al. which suggested that white matter is more prone to early diffusion lesion reversal [35]. White matter injuries in non-infarcted areas have been also described in cats [36] and rats [37]. In rats, vacuolization and pallor of the white matter were very marked after 24 hours and reflected the segmental swelling of myelinated axons, the formation of spaces between myelin sheaths and axolemma, and astrocyte swelling. The authors concluded that although white matter is generally considered less vulnerable to ischemia than gray matter, it is in fact highly vulnerable with pathological changes in oligodendrocytes and myelinated axons appearing early and independent of neuronal perikaryal injury [37].

### Limitations

Despite our promising results, several limitations should be noted. One limitation is the constraints posed by quantifying motor scores with the NIHSS for upper and lower motor scores. Data presented in this study reflected our clinical practice, in which complex motor scores to quantify motor function, such as with the Fugl Meyer Scale or the 9-hole peg test, were not acquired. Moreover, the motor score (MOT = UL+LL) could not be used in a ROC analysis as it is difficult to interpret good prognosis (low MOT score) vs. poor prognosis (high MOT score). For example, a low MOT score (i.e. 3/8) could be due to a severely hemiplegic upper limb (3/4) and a perfectly functional lower limb (0/4).

The second limitation could be that the study was performed on thrombolyzed patients. Thrombolysis can reverse some ischemic damage and salvage the ischemic penumbra tissue within a short time window (which is less than 12 hours) [38]. However, as we acquired the diffusion data at 24 hours, our measurements reflect already infarcted tissue.

A third limitation was the high dropout rate. More than half of our original patient population was unable to complete the follow-up DTI. No significant differences in admission, NIHSS scores, age, or thrombolysis delay were observed in the included *vs*. excluded patients, suggesting patients were not selectively enrolled in the study. Nevertheless, the statistical power of our analyses decreased due to a smaller population size. Indeed, a higher inclusion rate may have been able to untangle the relations between MD and AD in the corona radiata with partial correlations.

Finally, while the dose of physical therapy was comparable among patients, the type (passive or active) depended on individual disability. The interaction between rehabilitation therapy and early diffusion changes and its effects on clinical outcome are unknown.

## Conclusion

Our findings support the idea that stroke damage to white matter pathways should be studied using axial diffusivity measures at early time points. Furthermore, all of our data were processed through an automated pipeline that transforms native FA, MD, and eigenvalue maps into a normalized space. This allows for a systematic computation of diffusion metrics in corresponding brain areas in patients regardless of interindivual differences. Finally, this method can be incorporated into clinical routine.

## Supporting Information

S1 Table Infarct-ROI percent overlap in the regions of interest. (DOC)

S1 Text Investigation of Tensor Directions of the Corona Radiata Region of Interest (ROI). (DOC)
