# Supplementary material for: Axial Diffusivity of the Corona Radiata at 24 Hours Post-Stroke: A New Biomarker for Motor and Global Outcome
Source: PLoS One. 2015 Nov 12;10(11):e0142910. doi: 10.1371/journal.pone.0142910 (PMC4642950; doi:10.1371/journal.pone.0142910)
Supplement: S1 Table — (DOC) [file pone.0142910.s001.doc]

**S1 Table. Infarct-ROI percent overlap in the regions of interest.**

| **Subject** | **CP** | **PLIC** | **CoRad** | **M1** | **gCC** |
| --- | --- | --- | --- | --- | --- |
| 1 | 0 | 0 | 0 | 0 | 0 |
| 2 | 0 | 0 | 0.1 | 0 | 0 |
| 3 | 0 | 0.6 | 2.6 | 0 | 0 |
| 4 | 0 | 7.0 | 0.5 | 0 | 0 |
| 5 | 0 | 0 | 0 | 0 | 0 |
| 6 | 0 | 51.8 | 39.4 | 0 | 0 |
| 7 | 0 | 1.2 | 2.3 | 0 | 0 |
| 8 | 0 | 0 | 4.7 | 0 | 0 |
| 9 | 0 | 36.1 | 0.9 | 0 | 0 |
| 10 | 0 | 0 | 0 | 0 | 0 |
| 11 | 0 | 0.19 | 0.1 | 0 | 2.3 |
| 12 | 0 | 0 | 0 | 0 | 0.2 |
| 13 | 0 | 1.3 | 13.5 | 21.3 | 0 |
| 14 | 0 | 0 | 0.6 | 0.5 | 0 |
| 15 | 0 | 18.0 | 10.8 | 0 | 0 |
| 16 | 0 | 20.0 | 21.0 | 0 | 0 |
| 17 | 0 | 0 | 0 | 0 | 0 |
| 18 | 0 | 54.8 | 82.7 | 89.7 | 69.1 |
| 19 | 0 | 0 | 2.6 | 1.4 | 0 |
| 20 | 0 | 0 | 18.4 | 1.7 | 0 |
| 21 | 0 | <0.1 | 5.2 | 0 | 2.7 |
| 22 | 0 | 0 | 2.3 | 0 | 2.7 |
| 23 | 0 | 2.2 | 0.2 | 0 | 0 |
| 24 | 0 | 18.4 | 8.0 | 0 | 0 |
| 25 | 0 | 5.0 | 6.7 | 0 | 0 |
| 26 | 0 | 28.7 | 35.7 | 0 | 0 |
| 27 | 0 | 0 | 11.6 | 1.5 | 0 |
| 28 | 0 | 0 | 8.8 | 0 | 0 |

CP = Cerebral Peduncles, PLIC = Posterior Limb of Internal Capsule, CoRad = Corona Radiata; M1 = Underlying white matter of the precentral gyrus, gCC = genu of Corpus Callosum.
