# Supplementary material for: Axial Diffusivity of the Corona Radiata at 24 Hours Post-Stroke: A New Biomarker for Motor and Global Outcome
Source: PLoS One. 2015 Nov 12;10(11):e0142910. doi: 10.1371/journal.pone.0142910 (PMC4642950; doi:10.1371/journal.pone.0142910)
Supplement: S1 Text — (DOC) [file pone.0142910.s002.doc]

**S1 Text. Investigation of Tensor Directions of the Corona Radiata Region of Interest (ROI)**

**Objective**

The corona radiata (CoRad) contains a myriad of directions of tracts and fiber crossings, which are hard to be resolved in a single voxel by diffusion tensor imaging (DTI). It is therefore crucial to determine the fiber populations existing in the CoRad region of interest (ROI) that are reflected in diffusion measures. Our original hypothesis is that the refined CoRad ROI would contain primarily descending motor fibers from the motor, pre-motor, and supplementary motor cortices. To confirm this idea, the primary eigenvector volumes generated by FSL were used to calculate the deviation of the vector from the z-axis as an angle in degrees. Projection fibers would be closely aligned with the z-axis, whereas association fibers would be mostly perpendicular to the z-axis.

**Materials and Methods**

In order to align all of the subject’s native scans, a rigid transformation with 6 degrees of freedom was estimated by registering each subject’s native fractional anisotropy (FA) map to the FMRIB58_FA standard space image using FMRIB’s Linear Image Registration (FLIRT) in FSL [1-2]. This rigid transformation was then applied to each patient’s native 3D primary eigenvector volume generated by the FDT Diffusion Toolbox in FSL (http://fsl.fmrib.ox.ac.uk/fsl/fslwiki/). This operation merely shifts the voxels to the desired end position but does not correct for the orientation of the vectors inside the voxels. In order to remedy this, the rotation part of the rigid transformation was applied to the vector coordinates at each voxel using nibabel in the python programming language (http://nipy.org/nibabel/). The x,y,z coordinates of the aligned eigenvector volume were then used to calculate a volume containing the spherical coordinate *θ* at all voxels, using the following equation


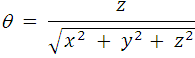


where the angle *θ* represents the deviation from the z-axis. Finally, the CoRad ROI of both hemispheres in MNI-space was projected into each patient’s aligned native volumes in a one step process using the reverse TBSS transformation with the aforementioned rigid transformation as a post-matrix, using FMRIB’s Non-linear Registration Tool (FNIRT) [3].

Normalized histograms for all of the angles *θ* (in degrees) in the transformed CoRad ROIs for each patient were calculated. Finally, an average normalized histogram was calculated representing the average distribution of angles in the corona radiata for all patients.

**Results**

Figure S1 shows an FA map of a representative subject after rigid alignment with the FA template, the transformed CoRad ROI, and the transformed primary eigenvector volume, along with the associated histograms and the average normalized histogram. The majority of the eigenvectors are oriented around 25° from the z-axis with a small spread in both the ipsi and contralesional hemisphere, most likely representing projection fibers originating from the motor cortices.

**S1 Fig. Axial Diffusivity Directions of the Corona Radiata**


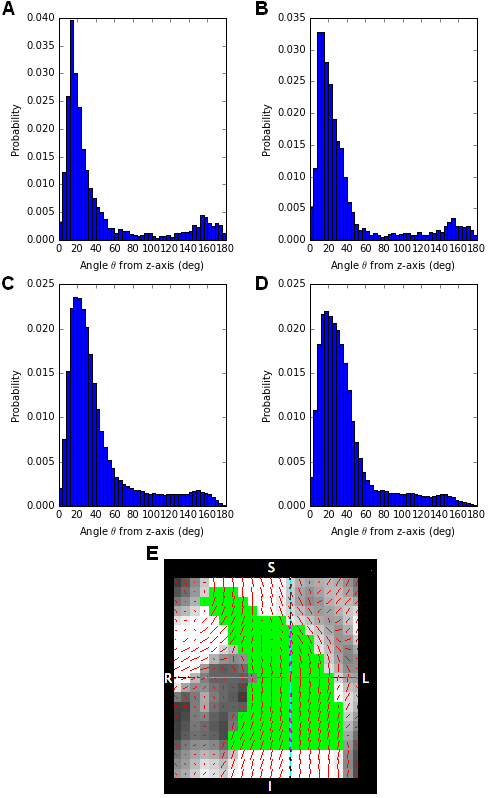


Normalized histograms of the (A) ipsi- and (B) contralesional corona radiata of a representative subject. Average normalized histograms of the (C) ipsi- and (D) contralesional corona radiata of the patient cohort. (E) FA map of the same representative subject with the corona radiata ROI overlaid in green and the orientation of the axial diffusivity eigenvalue in red.

There are very few fibers with orientations near the x-y plane around 90°, suggesting that the tensors do not capture association fibers through this ROI. Diffusion measures in the CoRad ROI reported in this article therefore most likely reflect damage to descending corticofugal fibers.

**References**

1. Jenkinson M, Smith SM. A global optimisation method for robust affine registration of brain images. Med Image Anal. 2001;5:143-156.

2. Jenkinson M, Bannister P, Brady M, Smith S. Improved optimization for the robust and accurate linear registration and motion correction of brain images. Neuroimage. 2002;17: 825-841.

3. Andersson JLR, Jenkinson M, Smith S. Non-linear registration, aka spatial normalisation. FMRIB technical report TR07JA2. 2010.
